# Feasibility of an interdisciplinary primary care-based intervention to improve care transitions for hospitalized patients with substance use disorders: The IntACT pilot randomized controlled trial protocol

**DOI:** 10.1371/journal.pone.0359281

**Published:** 2026-09-24

**Authors:** Michael A. Incze, Ulises Amaton, Justin D. Smith, Molly B. Conroy, Ingrid A. Binswanger, Stefan Kertesz, Valerie M. Vaughn, Adam J. Gordon

**Affiliations:** 1 Division of General Internal Medicine, Department of Medicine, University of Utah Health, Salt Lake City, Utah, United States of America; 2 Division of Health System Innovation and Research, Department of Population Health Sciences, University of Utah School of Medicine, Salt Lake City, Utah, United States of America; 3 Institute for Health Research, Kaiser Permanente Colorado, Aurora, Colorado, United States of America; 4 Colorado Permanente Medical Group, Denver, Colorado, United States of America; 5 Division of General Internal Medicine, University of Colorado School of Medicine, Aurora Colorado, United States of America; 6 Department of Health Systems Science, Bernard J. Tyson Kaiser Permanente School of Medicine, Pasadena, California, United States of America; 7 Birmingham, Alabama Veterans Affairs Medical Center, Birmingham, Alabama, United States of America; 8 University of Alabama at Birmingham Heersink School of Medicine, Alabama, United States of America; 9 Program of Addiction Research, Clinical Care, Knowledge, and Advocacy (PARCKA), Division of Epidemiology, Department of Internal Medicine, University of Utah School of Medicine, Salt Lake City, Utah, United States of America; 10 Informatics, Decision-Enhancement, and Analytic Sciences (IDEAS) Center, VA Salt Lake City Health Care System, Salt Lake City, Utah, United States of America; Public Library of Science, UNITED STATES OF AMERICA

## Abstract

**Background:**

Hospitalizations are common among individuals with substance use disorders (SUD) and represent crucial opportunities to engage patients in treatment. Quality efforts to increase SUD treatment access in hospital settings must be paired with interventions that support linkage to follow-up SUD and medical care. However, the best way to support patients with SUD during transitions of care after hospitalization is unknown.

**Methods:**

The IntACT pilot study is a single-site randomized controlled trial that aims to assess the feasibility and preliminary effectiveness of a primary care-based Interdisciplinary Addiction Care Transition (IntACT) team. IntACT deploys from a partnering primary care clinic to the hospital to meet patients with SUD while they are admitted, then follows them after discharge for four months to facilitate linkage to and retention in follow-up care. The pilot study will randomize 75 hospitalized patients with SUD to receive IntACT or Usual Care, assessing both implementation and effectiveness outcomes over a six month study period. Primary outcomes include implementation measures (e.g., reach, acceptability, feasibility, and cost) assessed using a mixed methods approach, as well as preliminary measures of effectiveness including successful linkage to follow-up medical and/or SUD care after hospitalization and retention in treatment. Secondary effectiveness measures include time to first post-discharge follow-up visit, substance use patterns, and acute care utilization (e.g., hospital readmissions). The IntACT study will also enroll 20 clinicians from hospital and outpatient settings who interact with IntACT while providing patient care to provide input on intervention feasibility, appropriateness, and acceptability. Study design and procedural elements will also be assessed for feasibility and acceptability.

**Discussion:**

Identifying promising interventions to support transitions to follow-up SUD and medical care following hospitalization is imperative to increase SUD treatment access and improve care quality. The IntACT pilot study evaluates the feasibility of a novel, potentially scalable team-based care transition intervention, gathering implementation data from both patients and clinicians who are exposed to the intervention. The results of this pilot study will inform intervention refinement for a future fully-powered clinical trial, as well as other implementation and research efforts supporting care transitions for people with SUD.

**Trial registration:**

Clinicaltrials.gov NCT07575373

## Background

Deaths attributable to the overdose public health crisis have cumulatively surpassed 700,000, with an American dying from drug overdose every 7.5 minutes in 2025 [1]. In 2022, opioid overdose was responsible for an estimated 3.1 million years of life lost in the US, [2] while alcohol directly contributes to approximately 178,000 deaths annually [3]. Despite the availability of effective pharmacotherapies, treatment for substance use disorders (SUD) is not accessed by the vast majority of individuals who could benefit [4, 5] As a result, expanding access to SUD treatment is a public health imperative for the US.

Hospitals have emerged as important venues for expanding access to SUD treatment. It is estimated that 1 in 9 hospitalized patients has a SUD, [6] and 25% of people with opioid use disorder have experienced a medical hospitalization in the past year [7]. Hospital-based SUD care models like addiction consult services and buprenorphine initiation protocols have enabled many hospitals to significantly expand access to inpatient SUD care initiation, particularly with medication treatment [8–11]. Despite the success of these models within the hospital, sustained treatment engagement after hospital-based medication initiation is modest, [8,12] and the best way to connect patients to long-term medical and SUD care after hospitalization remains unknown. Further, the period immediately following hospital discharge is associated with an acutely elevated risk of mortality for patients with OUD, [13,14] making identification of effective models to support patients through these transitions a pressing need.

While various post-hospitalization care transition models have been implemented on a small scale (i.e., within a single hospital or clinic), few have been rigorously studied [15,16]. Existing models include hospital-based addiction consult services, [8,9,17,18] discharge protocols for hospital teams, [19–22] transitional care interventions like peer navigation and care management, [23–27] and outpatient-based interventions like bridge clinics and post-discharge outreach [21,28–31]. Many of these interventions are designed to work within one part of the care transition spectrum, rather than across inpatient, transitional, and outpatient settings. For example, a hospital-based addiction consult team might initiate buprenorphine for opioid use disorder and assist with scheduling follow-up appointments, but once the patient leaves the hospital the consult team no longer provides support. Novel interventions that are designed to operate across inpatient and outpatient care settings with a single care team may improve care linkage and early retention in treatment. Whether these types of models are feasible and acceptable requires study through the lens of implementation science as well as using traditional effectiveness research.

Primary care is optimally suited to play a central role in SUD care transitions because of its geographic reach, team-based care models, greater accessibility relative to specialty SUD treatment, and expertise in chronic disease management. However, patients with SUD may face substantial barriers to transitioning to primary care-based treatment after hospitalization, including cravings, insurance limitations, lack of rapid access to care, unreliable transportation, and unstable housing. Furthermore, primary care clinicians’ (PCP) readiness to provide SUD care is affected by inadequate training, support, and even stigma. Models of care that integrate substance use expertise and interdisciplinary support into primary care environments may simultaneously improve confidence and quality of primary care-based SUD treatment while partnering with acute care settings like hospitals to strengthen care transitions to long-term, comprehensive follow-up [32].

This study’s objective is to evaluate the feasibility of integrating an Interdisciplinary Addiction Care Transition (i.e., IntACT) Team – including a Board-certified addiction specialist, a nurse care manager, and a peer support specialist – into a primary care setting to improve care transitions and retention in SUD treatment following medical hospitalization. Our central hypothesis is that adding interdisciplinary support will be feasible, acceptable, and will improve linkage to and retention in primary care and SUD treatment.

## Methods

### Objectives

**Fig 1:**
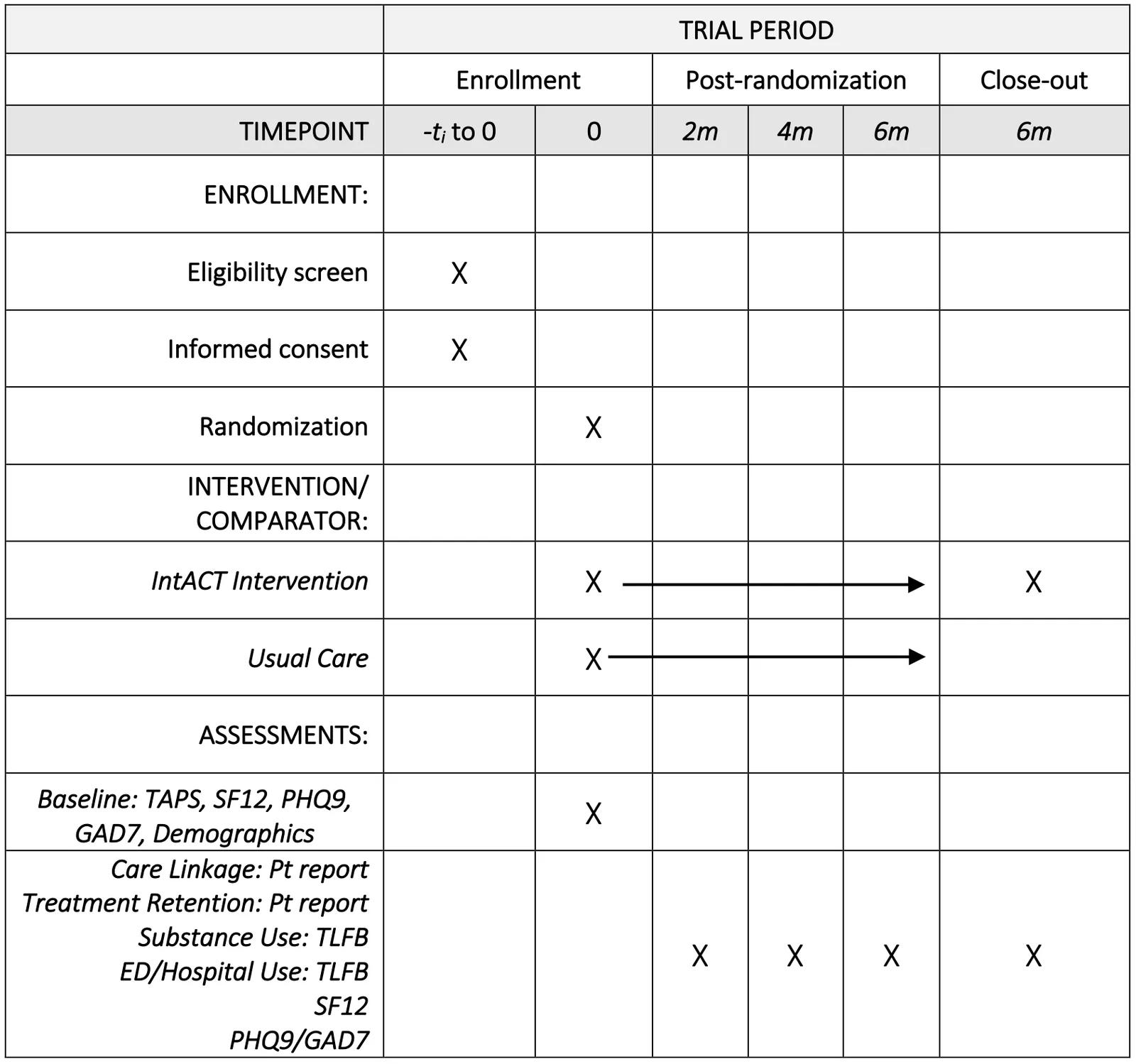
Participant timeline of enrollment, interventions, and assessments. Abbreviations: SF-12 = 12-Item Short Form Survey; TAPS = Tobacco, Alcohol, Prescription Medication, and Other Substances Tool; PHQ-9 = Patient Health Questionnaire-9; GAD-7 = Generalized Anxiety Disorder-7; TLFB = Timeline Follow-Back; IntACT = Interdisciplinary Addiction Care Transition team.

The primary objectives of the IntACT pilot randomized controlled trial (RCT) are to: 1) evaluate the implementation of the IntACT team-based intervention, and 2) obtain preliminary measures of effectiveness related to post-hospitalization care linkage and engagement in follow-up treatment, compared to Usual Care (Fig 1). This pilot study will inform the refinement of intervention components and implementation strategies to be studied in a subsequent fully-powered Hybrid Type 2 Implementation-Effectiveness RCT [33,34].

The IntACT trial’s secondary objectives are to measure the intervention’s effects on: 1) time from discharge to first completed follow-up medical or SUD visit, 2) substance use patterns, and 3) rates of acute care utilization (i.e., hospital readmission, emergency department [ED] or urgent care visits). Including a broad range of effectiveness outcomes will help to determine the feasibility of the study’s assessment approach and outcomes selection for a future fully-powered trial.

### Study design

The IntACT pilot study is a single-site RCT that will enroll patients with SUD during an index hospitalization and randomize them to receive IntACT or Usual Care over an intervention period of 4 months (Fig 1) [35]. Randomization is unbalanced, with a ratio of 3:2 favoring the intervention arm over Usual Care. This randomization scheme is intended to prioritize data collection about the feasibility and implementation of IntACT by favoring assignment to the intervention arm. Additionally, we expect IntACT to be superior to Usual Care and therefore have an ethical rationale for more participants receiving the intervention. Finally, unbalanced randomization has only slightly less power than a balanced design when the absolute differences between the groups are small [36,37]. The randomization sequence will be developed by an impartial third party at the University of Utah Clinical and Translational Science Institute using random permuted blocks and embedded into a secure online platform (REDCap). The allocation sequence will be concealed until the time of assignment to IntACT or Usual Care. While the nature of the intervention precludes masking for patients and clinicians, statisticians will be masked to study arm assignment.

### Recruitment and eligibility

Recruitment will occur at a single 425-bed tertiary academic medical center. A total of 75 patients will be recruited. Patient inclusion criteria include: 1) aged 18 years and older, 2) hospitalized at the study site for any medical reason, and 3) Electronic Health Record (EHR) documented diagnosis of any SUD. Exclusion criteria are: 1) currently incarcerated, and 2) Tobacco Use as the only documented SUD. Potential participants will be identified through a combination of clinician referral (i.e., the study will be advertised to hospital clinicians and staff, and hospital care teams and the addiction consult service can refer patients to the study) and prescreening. The prescreening process will consist of the IntACT research coordinator reviewing a curated list of daily hospital admissions filtered to contain new medical admissions with a documented SUD. Admission can be for any medical cause. Patients from the prescreening list that appear to meet eligibility criteria after EHR review will be approached and screened for enrollment.

Additionally, 20 clinicians who interact with IntACT will be recruited from both inpatient and outpatient settings to assess IntACT’s feasibility and acceptability. Clinician inclusion criteria include: 1) aged 18 years and older, 2) provided direct clinical care in any capacity to enrolled patients either in the hospital or in the outpatient setting (e.g., hospitalist, PCP, specialist/consultant), and 3) interact with the IntACT team in a clinical/shared patient care capacity (e.g., coordinated discharge planning). Exclusion criteria include an inability to complete surveys and/or interviews in English or Spanish. Potential participants will be identified through review of clinical EHR notes for enrolled patients and through word of mouth from the IntACT clinical team.

### Intervention

Participants randomized to IntACT will receive usual inpatient and outpatient services plus an interdisciplinary addiction care transition team consisting of a Board-certified addiction specialist, a nurse care manager, and a peer support specialist. The IntACT intervention provides: 1) in-hospital discharge planning support, 2) proactive post-discharge outreach and care coordination, 3) intensive care management and peer support, and 4) interim SUD and medical care coordination for up to four months post-randomization, with the goal of facilitating transition to longitudinal community-based care.

Upon randomization to the IntACT study arm, a member of the IntACT team (either peer navigator, clinician, or care manager) will deploy in person to the hospital to meet the participant, introduce their role, and exchange contact information. Initial contact will be made within 2 business days of randomization. The IntACT team member will discuss potential discharge needs with the participant and offer to facilitate any needed follow-up care at a partnering primary care/addiction medicine clinic or at a community site of the participant’s choosing. A one-page contact sheet will be provided to the patient with a IntACT team cell phone number and contact information for both our peer support specialist and care manager during the initial visit. If the patient leaves the hospital after randomization and before a member of the IntACT team can meet them, remote (e.g., telephone, text) outreach will be provided to complete the initial clinical meeting.

The IntACT team will continue to communicate with the patient and the care team ad hoc during the remainder of the hospitalization to address any care coordination needs that arise. At weekly IntACT clinical team meetings, the team will review the new participant’s hospital course, medications, and discharge needs. Ensuing discussion will focus on how best to leverage the interdisciplinary IntACT team to improve post-discharge care, including linkage to community supports, addressing biopsychosocial barriers to follow-up, and ensuring appropriate access to SUD and medical care.

Within two business days of discharge, the IntACT team will reach out to the patient, inquiring about symptoms and challenges faced after discharge, reviewing upcoming appointments and discharge medications, and assessing post-discharge substance use needs. If the team is unable to reach the participant, they will keep trying at least three times per week for the first three weeks after discharge. While follow-up visit frequency will be individualized, most people will follow up every 1–2 weeks via appointments at the partnering clinic or at another clinic/program of the participant’s choosing. Between-visit care through telephone and text outreach will be facilitated by IntACT team members. There are no additional clinical visits required of the patient to receive care through IntACT.

Participants randomized to Usual Care will receive standard services routinely available at the study site, including a hospital addiction consult service, discharge planning by hospital medical and social work teams, and referrals/follow-up options based on insurance and patient preference. Usual Care participants will not receive the structured IntACT components provided in the intervention arm. However, because IntACT team members work in clinical roles within the study site, participants in both arms may receive care that includes members of IntACT. If a Usual Care participant independently establishes care with the partnering primary care/addiction medicine clinic or other services (e.g., peer support) through standard referral pathways, this will be permitted and will be captured as part of outcome assessment.

After four months, the official intervention period will stop. Participants in both groups will still be able to continue care with their current medical and SUD treatment teams.

### Partnership of people with lived substance use experience in the intervention design

Individuals with lived substance use experience informed the design of the IntACT intervention both directly and indirectly. Prior to designing the intervention, we conducted qualitative interviews with people with lived SUD experience who had recently gone through a hospitalization about their care transition experiences [38]. Their expertise helped to formulate key elements of the intervention, including the outpatient-to-inpatient deployment of a primary care-based team. We also conducted a series of focus groups with both clinicians and people with lived substance use experience about key barriers and facilitators to SUD care transitions, components of an ideal care transition intervention, and priorities for future research [39]. Finally, the intervention team employs a peer support specialist, who provided direct formative feedback on IntACT’s approach, implementation, and evaluation during weekly pre-implementation meetings.

### Underlying conceptual framework for implementation

**Fig 2:**
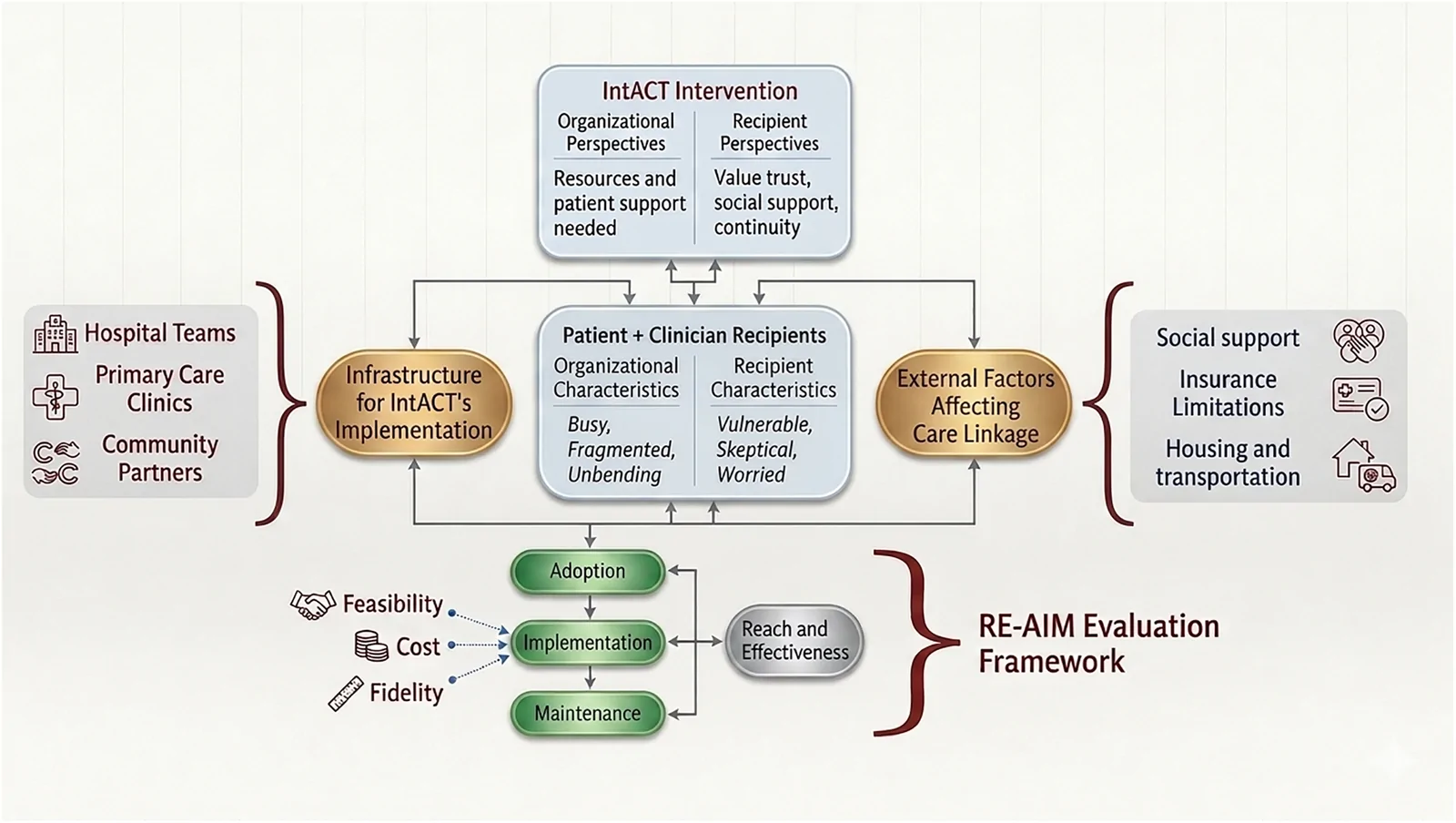
The PRISM and RE-AIM Implementation Frameworks adapted to the IntACT Study.

Two complementary conceptual frameworks will guide the implementation of the IntACT intervention: the Practical, Robust Implementation and Sustainability Model (PRISM) determinants framework and the Reach, Effectiveness, Adoption, Implementation, and Maintenance (RE-AIM) evaluation framework (Fig 2) [40,41]. RE-AIM specifically informs the evaluation plan for IntACT, including measures of reach, effectiveness, and implementation (e.g., feasibility). PRISM provides an overarching framework that accounts for the influence of real-world contextual factors such as clinician and staff attitudes and environmental/time constraints on intervention implementation.

**Fig 3:**
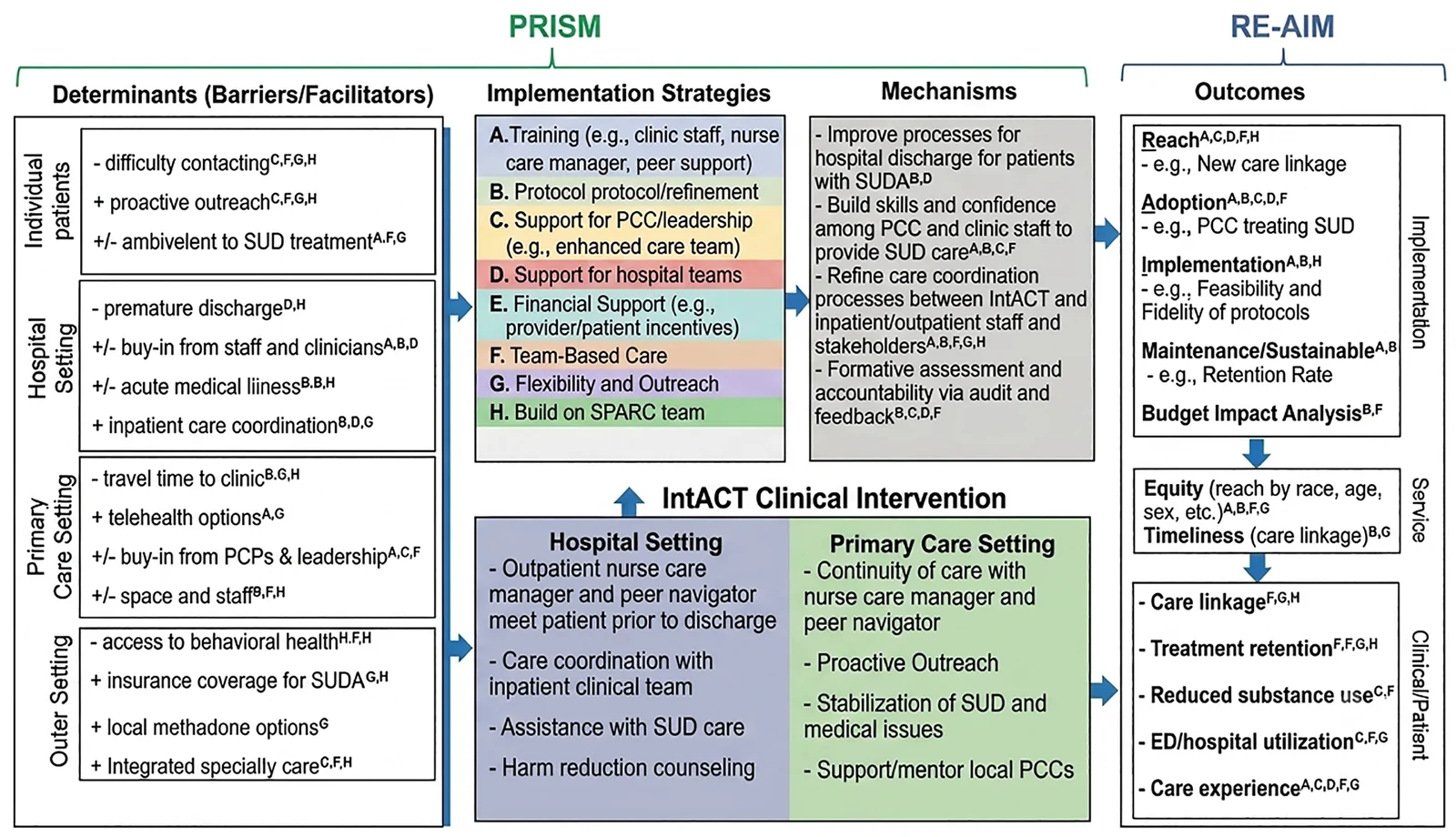
Implementation Research Logic Model for the IntACT Intervention. Superscripted letters represent how implementation strategies are carried throughout the model to address specific barriers and inform mechanisms and outcomes design.

For the IntACT study, these complementary frameworks are integrated into an Implementation Research Logic Model (IRLM), [42] which is an organizational tool designed to synthesize implementation science frameworks into a single instrument that can be used to guide planning, execution, reporting, analysis, and reproducibility of implementation research (Fig 3). The IRLM has been used in multiple chronic disease implementation trials, highlighting its adaptability as an instrument [43–45]. In this study, the IRLM serves as a scaffold to integrate PRISM and RE-AIM for the planning, implementation, and evaluation of IntACT.

### Procedures and assessments

Key study-related procedures include participant enrollment and completion of iterative outcome assessments during and after the 4-month intervention period. These are outlined in Table 1.

**Table 1:** Assessment timeline for patient participants.

Study Procedure	Baseline	Month 2	Month 4	Month 6
Eligibility confirmation and consent	X			
Baseline survey (demographics and substance use history questionnaire)	X			
Study visit/ follow-up contact	X	X	X	X
SF-12	X	X	X	X
TAPS (Baseline only)	X			
PHQ-9	X	X	X	X
GAD-7	X	X	X	X
TLFB (Substance Use and ED/Hospital Encounters; Follow-up visits only)		X	X	X
Qualitative interview (n = 15; IntACT arm only)				X
Adverse event assessment		X	X	X

*Abbreviations: SF-12 = 12-Item Short Form Survey; TAPS = Tobacco, Alcohol, Prescription Medication, and Other Substances Tool; PHQ-9 = Patient Health Questionnaire-9; GAD-7 = Generalized Anxiety Disorder-7; TLFB = Timeline Follow-Back; IntACT = Interdisciplinary Addiction Care Transition team.*

If a potential participant meets eligibility criteria during the screening process, the research coordinator will explain the study objectives, procedures, and the randomized nature of the trial; assess interest in participation; and conduct a formal written informed consent process for those who wish to enroll. Once consented, participants will complete a baseline survey to finalize enrollment. Individuals unable to provide informed consent due to acute intoxication, withdrawal, medical illness, or psychiatric symptoms causing decisional impairment will not be enrolled. Trained research staff will re-approach these patients a minimum of 4 hours later to reassess their ability to consent to study participation. This assessment will be based on gestalt of the patient’s clinical status (e.g., intubation, sedation) and ability to answer questions in a linear and comprehensible manner. Once a participant is enrolled in the study, they will be randomized to receive the intervention or Usual Care.

For the clinician cohort, potentially eligible clinicians (i.e., those identified as providing clinical care to intervention patients) will be contacted by study staff either verbally or via secure institutional email and screened for eligibility based on inclusion/exclusion criteria. If eligible, research staff will assess interest in participation and conduct a formal informed consent process for those who wish to enroll. To minimize any possibility of undue influence, clinician recruitment will be conducted by the research coordinator or study staff, not by supervising physicians or direct clinical colleagues.

In addition to recruitment and enrollment, study procedures also involve iterative assessment of primary and secondary study outcomes (Table 1). Outcome assessment will be led by study staff and take place at scheduled in-person study visits or, if necessary, remotely over telephone or videoconferencing. These assessments will consist of a series of surveys and occur at baseline, 2, 4, and 6 months after randomization. Patient participants will be compensated $35 for completing each of the first three assessments (i.e., baseline, 2 month, 4 month) and $75 for completing the final 6-month close-out visit (total potential earnings $180). Purposive sampling will be used to recruit fifteen intervention participants who completed the final close-out visit to participate in a 45-minute semi-structured interview about their experience with IntACT. Compensation for completing the interview will be $75. If a participants misses study assessment visits, they will still be invited to conduct a study close-out visit at six months and complete the end of study survey.

Enrolled clinicians will complete an implementation survey after each exposure to IntACT (e.g., IntACT assists with discharge planning for a patient that they are caring for in the hospital). These surveys will use validated tools to assess the feasibility, acceptability, and appropriateness of IntACT, its specific components, and its implementation strategies from a clinician’s perspective. Purposive sampling will be used to recruit a subset of twelve clinicians who had extensive interactions with IntACT to complete semi-structured interviews further exploring experiences and perceptions related to the intervention. IntACT team members (n = 3) will be surveyed and interviewed one time at the end of the study period using these same assessment tools. IntACT team members will also complete a fidelity checklist one time for each new intervention patient enrollment documenting the clinical and psychosocial services provided. Clinician participants will be compensated $30 for completing each survey and an additional $75 for completing a 45-minute semi-structured interview.

### Outcomes

The outcomes of the IntACT trial are represented in Table 2. Primary implementation outcomes include feasibility, acceptability, appropriateness, reach, and cost. Clinician participants and IntACT team members will complete validated survey instruments and semi-structured interviews evaluating the feasibility, acceptability, and appropriateness of the IntACT intervention. These assessments will assess the implementation of IntACT overall, along with its specific intervention components and implementation strategies from the IRLM (Fig 2). Reach will be assessed using administrative data (e.g., EHR, study records) denoting the number of clinicians and community follow-up sites that interact with IntACT. Costs will consist of estimated intervention costs (e.g., team member salary and effort, cell phone).

**Table 2:** IntACT pilot study key outcomes.

Pilot Trial Measures				
Variable	Measure	Data Source	Method	Frequency
**Implementation Outcomes**				
Acceptability	4-item Acceptability of Intervention Measure, Interviews	CT, IT, PT	S, Qual	End of Exposure
Appropriateness	4-item Intervention Appropriateness Measure, Interviews	CT, IT, PT	S, Qual	End of Exposure
Feasibility	4-item Feasibility of Intervention Measure, Study Enrollment	CT, IT, PT	S, Qual	End of Exposure
Reach	Number of Pt and PCP who participate in and complete the study	EHR/admin	S, Qual	Monthly
Cost	Costs of intervention staffing, training, and infrastructure	Admin	Admin	Quarterly
**Primary Effectiveness Outcomes (compared between intervention and control groups)**				
Care Linkage	% of participants attending an outpatient visit within 14 days of discharge	EHR/admin	LR	Once/patient
Treatment Retention	% of patients with evidence of SUD treatment engagement (e.g., active prescription, visit with associated SUD diagnosis within past month)	EHR/admin	LR	At 2, 4, and 6 months
**Secondary Effectiveness Outcomes (compared between intervention and control groups)**				
Time to First Visit	Number of days elapsed between discharge and first primary care appt	EHR/admin	LR	Once/patient
Acute Care Utilization	# of ED, hospital, or urgent care visits during the 6 months preceding and 6 months following enrollment	PT	S, Qual	At 2, 4, and 6 months
Substance Use	Self-reported frequency and amount of substance use	PT	S, Qual	At 2, 4, and 6 months

**Abbreviations**: CT = clinical team (PCP, hospitalists, social workers). IT = IntACT team members. S = Survey. Qual = qualitative interviews or focus groups, Admin = administrative data. PT = patient. EHR = electronic health record. LR = logistic/linear regression

Primary effectiveness outcomes will be compared between intervention and control groups and include: 1) the percentage of participants who attend a follow-up visit (e.g., primary care, specialty care, SUD care) within 14 days of discharge, and 2) the percentage of participants with evidence of SUD care engagement at 2, 4, and 6 months following discharge, including attendance at scheduled visits and/or documentation of medication for substance use disorder receipt. Secondary effectiveness outcomes include: 1) time to first follow-up visit after hospital discharge; 2) self-reported substance use; and 3) acute care utilization including hospital readmission, ED visits, and urgent care visits. These outcomes will be assessed using a combination of administrative data review and patient self-report using the Timeline Followback method, an established framework for assessing recent substance use patterns [46,47].

### Statistical analysis

Consistent with National Institutes of Health recommendations for pilot and feasibility studies, this trial is not designed for statistical power to definitively test effectiveness hypotheses [48]. Quantitative data related to implementation will be presented descriptively. Linear and logistic regression modelling will be used, as appropriate, to compare effectiveness outcomes between the control and intervention groups in order to test the feasibility of our assessment and analytic strategies (Table 2). Complete case analysis will be used as a primary approach to missing data. If there is a large amount of missing data, multiple imputations will be used to mitigate the effects of missing data.

Semi-structured interviews related to IntACT’s implementation will be recorded, transcribed and de-identified. Subsequently, the transcripts will summarized and deductively analyzed according to the domains of the PRISM implementation framework using a Rapid Qualitative Analytic approach [49–51]. Qualitative themes pertaining to intervention implementation will be identified from the summaries and compared with quantitative data to identify cross-cutting themes related to intervention acceptability, appropriateness, and feasibility. Mixed methods research frameworks (e.g., joint display) will be used to draw inferences from the combined quantitative and qualitative implementation data [52,53].

### Safety monitoring

This single-site, minimal-risk study does not require a formal Data and Safety Monitoring Board (DSMB). Study staff will monitor participants for adverse events potentially related to the intervention or research procedures, including confidentiality breaches and emotional or mental health distress during care coordination or data collection, as well as serious clinical outcomes (e.g., ED visits, hospitalizations, overdose, death) using the EHR and patient self-report during each study visit. Events that meet IRB reporting criteria will be reported according to institutional policy, and study procedures will be modified or paused if emerging safety concerns are identified.

### Ethics and dissemination

All study activities were approved by the University of Utah Institutional Review Board (#00195935). Study activities will be carried out in accordance with the ethical standards set forth in the Declaration of Helsinki. The results of this study will be disseminated in peer reviewed manuscripts.

### Trial status

The initial protocol for the IntACT trial received IRB approval in January 2026. The study and intervention teams were finalized in March 2026. The trial protocol was registered on Cliincaltrials.gov on May 11, 2026. Recruitment is scheduled to begin on June 21, 2026. We anticipate that enrollment will be complete by July 31, 2027. Data collection will be completed by February 28, 2028. Trial results are expected by May 31, 2028. This manuscript was based on the IRB-approved protocol (version 2.0).

## Discussion

This paper describes the study design and protocol for a pilot RCT evaluating a novel primary care-based care transition intervention for hospitalized patients with SUD. The IntACT intervention holds promise to address an important gap in SUD care – connecting individuals to longitudinal SUD and medical treatment following a hospitalization. Currently there are few rigorously studied models to support patients with SUD during post-hospitalization care transitions, leading to a dearth of guidance for health systems seeking to implement SUD care transition supports tailored to their respective environments. Given the high prevalence of SUD among hospitalized patients nationwide, identifying models that are effective and adaptable to a range of healthcare settings could have major implications for future research, clinical care, and health policy.

A strength of the IntACT model is its integration within primary care. Primary care-based interventions have a strong potential for scalability given the geographic reach of primary care nationwide. The team-based design of IntACT is also well-suited to modern primary care settings, which have increasingly prioritized the implementation of team-based care models [54,55]. Further, core features of IntACT, such as care management and remotely-delivered medical care, are already ingrained into many US primary care settings. However, integrating SUD treatment into primary care settings has proven challenging, with few primary care clinicians prescribing medications for SUD like buprenorphine [56–58]. Previous studies have identified factors such as a lack of local mentorship and support as factors that influence SUD care adoption [59–61]. Whether an integrated addiction care team co-managing new patients with SUD will help to overcome these barriers is unknown. The IntACT study includes a mixed methods assessment of IntACT’s acceptability and implementation among both hospital-based and outpatient clinicians. These measures can yield key information aimed at enhancing IntACT’s integration with primary care to inform intervention adaptation for future large-scale studies that include more diverse practice settings.

Another strength of this study is that the intervention spans care settings that are often siloed during post-hospitalization care transitions [15]. While the IntACT team is based in primary care, the intervention begins with in-reach to meet enrolled participants while they are still hospitalized. This step enables one clinical team to provide continuity of care before discharge, during the immediate post-hospitalization period, and then longitudinally for the following four months. There are numerous theoretical advantages to this approach, including providing interdisciplinary support during discharge planning, establishing rapport and exchanging accurate contact information with patients prior to discharge, and allowing for stronger lines of communication between inpatient and outpatient care settings. However, these advantages come at the cost of dedicated effort required to mobilize to the hospital and provide intensive care coordination after discharge. The IntACT study includes measures of feasibility that include cost estimates and post-discharge acute care utilization that will help to elucidate the financial sustainability of the model and which can inform future adequately-powered cost-effectiveness studies.

Finally, the team-based design of IntACT is a key feature that will be evaluated in this pilot study. Interdisciplinary teams bring complementary expertise that can confer synergy to complex clinical episodes like SUD care transitions. However, there are also additional investments required by health systems to support staff, training, and infrastructure (e.g., cell phone) for team-based care. The IntACT study includes both quantitative and qualitative assessments with intervention team members, clinicians who interact with the intervention, and patient participants to learn about the feasibility, unique benefits, and required investments for a team-based care transition intervention. The cost analysis will add another element of assessment to generate hypotheses about the relative benefits and costs of team-based models that can be studied in future comparative effectiveness studies with other types of interventions such as individual (e.g., a single care manager) models. The study’s use of established implementation science frameworks like PRISM and RE-AIM provide tools to contextualize our findings and inform conclusions about the implementation of team-based care to support SUD care transitions in general medical settings.

### Limitations

There are several limitations with this pilot study. By design, the study is conducted at a single-site involving a sample that lacks statistical power to generate definitive conclusions about effectiveness. Rather, this study is designed to assess the feasibility of intervention implementation and study design to inform future research. A second limitation is that the study relies predominantly on subjective assessment instruments such as patient recall, survey responses, and interviews, which are prone to social desirability bias and other potential confounders. However, the study predominantly uses validated survey instruments for quantitative assessment, and complementary qualitative data provides analytic depth to inform preliminary conclusions about the intervention. Finally, the composition of the IntACT team, which includes a Board-certified addiction specialist, a nurse care manager, and a peer support specialist, may not be generalizable to all practice settings. The IntACT study intends to lay the groundwork for future research on team-based models that can be adapted to diverse practice settings using locally-available personnel.

## Conclusion

The IntACT study is a single-site pilot RCT that will evaluate the feasibility and preliminary effectiveness of a novel primary care-based care transition intervention for hospitalized individuals with SUD. Given the pressing need to expand access to SUD treatment in the US, identifying promising interventions that proactively identify patients with SUD in high prevalence settings and facilitate linkage to longitudinal, comprehensive healthcare and SUD treatment is a crucial objective. The results of this pilot study can inform future broad implementation and evaluation of interventions designed to enhance care for hospitalized individuals with SUD.

## Abbreviations

SUD: Substance Use Disorder
PCP: Primary Care Clinician
IntACT: Interdisciplinary Addiction Care Transition
RCT: Randomized Controlled Trial
EHR: Electronic Health Record
IRLM: Implementation Research Logic Model
IRB: Institutional Review Board
SF-12: 12-Item Short Form Survey
TAPS: Tobacco, Alcohol, Prescription Medication, and Other Substances Tool
TLFB: Timeline FollowBack
PRISM: Practical, Robust Implementation and Sustainability Model
RE-AIM: Reach, Effectiveness, Adoption, Implementation, and Maintenance Framework
